# Vitamin D Receptor Gene *Fok*I Polymorphism Contributes to Increasing the Risk of Tuberculosis: An Update Meta-Analysis

**DOI:** 10.1097/MD.0000000000002256

**Published:** 2015-12-28

**Authors:** Liling Huang, Cunxu Liu, Guangfu Liao, Xiaobing Yang, Xiuwen Tang, Jingjie Chen

**Affiliations:** From the Department of Clinical Laboratory (LH, XY); Department of Tuberculosis (CL); Department of Central Laboratory (GL, XT); Department of Science and Education, Longtan Hospital of Guangxi Zhuang Automomous Region, Liuzhou, Guangxi, People's Republic of China (JC).

## Abstract

The association between vitamin D receptor (VDR) *Fok*I polymorphism and tuberculosis (TB) risk remains a matter of debate. Potential selection bias exists in most studies using HIV-positive TB patients.

An update meta-analysis was carried out to derive a more reliable assessment of the association between *Fok*I polymorphisms and TB risk, especially in HIV-negative TB patients. All major databases from inception to June 2015 were searched for all publications that studied the association between *Fok*I polymorphism and TB risk. The odds ratios (ORs) and the corresponding 95% confidence intervals (CIs) were calculated according to the frequencies of genotypes.

In total, 32 studies with 4894 cases and 5319 controls were included in this meta-analysis. In the overall analysis, the estimated OR was 1.34 (95% CI=1.091–1.646, *P* = 0.005) in the best genetic model (recessive model, ff vs fF+FF) with moderate heterogeneity (*I*^*2*^ = 32.2%, *P* = 0.043). In the subgroup analysis stratified by HIV status, significant associations were found only in the HIV-negative TB group (OR = 1.60, 95% CI = 1.180–2.077, *P* = 0.002; *I*^*2*^ = 29.5%, and *P* = 0.141 for heterogeneity). In the subgroup analysis stratified by ethnicity, significant associations were found in the Asian group (OR = 1.65, 95% CI = 1.205–2.261, *P* = 0.002; *I*^*2*^ = 43.9%, and *P* = 0.024 for heterogeneity), but not in the Caucasian group (OR = 1.09, 95% CI = 0.762–1.547, *P* = 0.649; *I*^*2*^ = 0.0%, and *P* = 0.740 for heterogeneity) and African group (OR = 0.99, 95% CI = 0.726–1.341, *P* = 0.934; *I*^*2*^ = 43.9%, and *P* = 0.024 for heterogeneity).

This meta-analysis confirms that VDR *Fok*I polymorphism contributes to the risk of TB, especially in HIV-negative TB patients and in the Asian group. Further studies are required to clarify the role of the *Fok*I polymorphism in HIV-positive TB and in other ethnic groups.

## INTRODUCTION

Tuberculosis (TB) is a global public health problem and remains a great burden throughout the world.^1^ The risk of developing TB ranges from 5% to 10% after infection by *Mycobacterium tuberculosis* (MTB) for individuals, and only a minority of individuals develops clinical disease, even though infected with virulent mycobacteria. Other factors, such as environmental and genetic factors, HIV infection, and diabetes, also play important roles in the process.^2–5^ Likewise, genetic factors are important in determining susceptibility and resistance to MTB and are considered related to the susceptibility to TB.^5,6^

Vitamin D is now considered to be a key factor in the body's defense against TB, mediated by binding to the vitamin D receptor (VDR) in monocytes, macrophages, and lymphocytes.^7,8^ The VDR gene is located in the chromosomal 12q13 region, and there are 4 classically typed single-nucleotide polymorphisms (SNPs), *Fok*I, *Bsm*I, *Apa*I and *Taq*I, which were studied intensively for association with various human traits and were reported to affect risk of variousdiseases.^9^ The *Fok*I restriction site defines an SNP (rs10735810, C to T) in the first of 2 potential translations—initiation start sites for VDR mRNA. The VDR protein synthesizes full-length (427 amino acids) in the alternate allele form (ATG) (designated f) and has 3 more amino acids than the VDR encoded by the common allele form (ACG) (designated F). The *Fok*I restriction site is a functional polymorphism of the VDR gene.^10^ The polymorphisms of *Fok*I can alter the amount of VDR produced ^9,11^ and are related to plasma vitamin D levels in TB patients.^12^

To date, the polymorphisms of *Fok*I have been studied in relation to the risk of TB in many populations; however, the results remained contradictory.^10,13–15^ Recently, Chen et al^16^ and Sun and Cai^17^ carried out meta-analyses focusing on the associations between *Fok*I polymorphisms and TB risk; these 2 meta-analyses missed many studies.^12,18–23^ Moreover, HIV infection status should be adjusted in studies focused on genetic susceptibility to TB since TB is the frequent major opportunistic infection in HIV-infected patients.^24^ Thus, we carried out an update meta-analysis to derive a more reliable assessment on the association between *Fok*I polymorphisms and TB risk, especially in HIV-negative TB patients.

## METHODS

The Preferred Reporting Items for Systematic Reviews and Meta-Analyses (PRISMA) statement was used in the process of the meta-analysis (Table S1).^25^

### Search Strategy and Study Selection

A search of the medical literature was conducted using the Embase, PubMed, and Cochrane Library databases through June 30, 2015. The search terms were used as follows: *vitamin D receptor* or *VDR* in combination with *polymorphism*, *polymorphisms*, and *mutation* or *variant* in combination with *tuberculosis* or *TB*. Two investigators (LH and XY) conducted an extensive literature search independently for all publications. Articles in reference lists were also hand-searched and authors of trial reports published only as abstracts were contacted and asked to contribute full datasets or completed papers. There were no language restrictions and only human studies were searched.

Case-control studies with enough data to calculate odds ratio (OR) were included in our study. We excluded duplicate studies or studies containing overlapping data. Family-based studies were also excluded.

### Data Extraction

All data were extracted independently by 2 investigators (LH and XY). The following clinical data were extracted from eligible studies: the baseline characteristics, such as the first author's name, publication year, country, ethnicity, total sample size, genotyping method, and source of control group, and details of TB types and genotype frequencies of cases and controls. Hardy-Weinberg equilibrium (HWE) was calculated from genotype frequencies of controls. Investigators would try to contact the author to get the original data if the literature could not provide sufficient data. A third reviewer (JC) resolved any discrepancies when the abovementioned reviewers disagreed.

### Statistical Analysis

In this study, we considered f is the increasing or risk allele; therefore, an allelic model (f vs F), a codominant model (ff vs FF, fF vs FF), a dominant model (ff+fF vs FF), and a recessive model (ff vs fF+FF) are accessed by calculating the unadjusted odds ratios (ORs) and the corresponding 95% confidence intervals (CIs) according to the frequencies of genotypes. To avoid the problem of multiple comparisons, we applied the method for meta-analysis of molecular association studies to dictate the best genetic model.^26^

Heterogeneity was assessed with a *χ*^2^*Q* test and *I*^2^ statistics. The heterogeneity was significant if *P*_*Q*_ < 0.1 or *I*^*2*^ > 50%, and a random-effects model was conducted using the DerSimonian and Laird method. Otherwise, the fixed-effects model (the Mantel-Haenszel method) was performed.^27,28^ A subgroup analysis of ethnicity was carried out considering that the same gene polymorphism plays different roles in the risk of diseases among different ethnic subpopulations. HIV-negative TB patients who were studied were also considered a subgroup and pooled in this meta-analysis. Galbraith plots analysis was performed for further exploration of the heterogeneity.

HWE in the controls was tested with the *χ*^2^ test for goodness of fit, and a *P* value <0.05 was considered out of HWE. Sensitivity analysis was conducted to examine such influence by removing studies one by one and by recalculating the pooled OR and 95% CI. The Begg rank correlation method and the Egger weighted regression method were used to statistically assess publication bias.

Ethical approval was not necessary, as this study is a meta-analysis, which is based on the published data.

All the tests in this meta-analysis were conducted with STATA software (version 12.0; Stata Corporation, College Station, TX); *P* <0.05 indicated that the result was statistically significant.

## RESULTS

### Study Excluded and Characteristics of Included Studies

**FIGURE 1:**
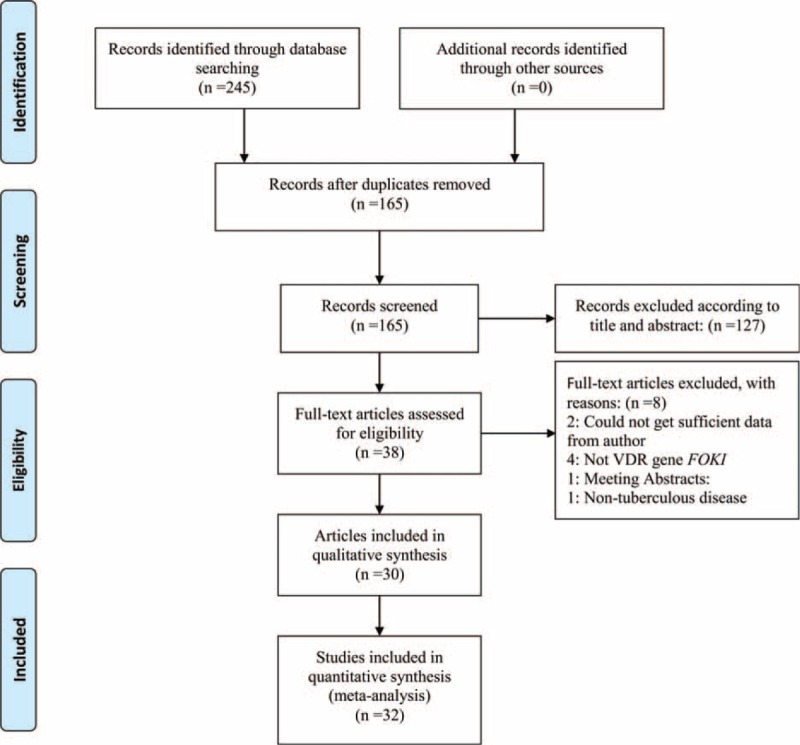
Flow diagram of included studies for this meta-analysis.

Thirty-eight articles were initially evaluated for the meta-analysis, of which 8 studies were excluded. Two studies were excluded because, even though an attempt was made to contact the study authors, no sufficient data were obtained.^29,30^ Four studies were excluded for not focusing on *Fok*I polymorphism.^31–34^ In addition, a meeting abstract ^35^ and a study about nontuberculous mycobacterial lung disease ^36^ were also excluded. The study by Alagarasu et al^13^ was separated into 3 studies for different TB types and HIV status. Finally, 32 studies with 4894 cases and 5319 controls met inclusion criteria. Details of the study flow are documented in Figure 1.

Table 2 shows a summary of the characteristics of the included studies. There were 18 studies involving Asians,^13–15,19,21–23,37–45^ 8 studies involving Caucasians,^12,18,43,46–50^ and 6 studies involving Africans.^20,51–55^ Fourteen studies included HIV-negative TB patients,^10,13–15,19,22,37,39,45,47,50,51,53,56^ but only the study by Alagarasu et al^13^ included HIV-positive TB patients, and the other 16 studies did not offer detailed information. The genotype distributions among the controls of all studies were consistent with HWE, with the exception of 3 studies.^39,44,49^ TB types, genotyping methods, and genotype numbers are shown in Table 2.

**TABLE 2:**
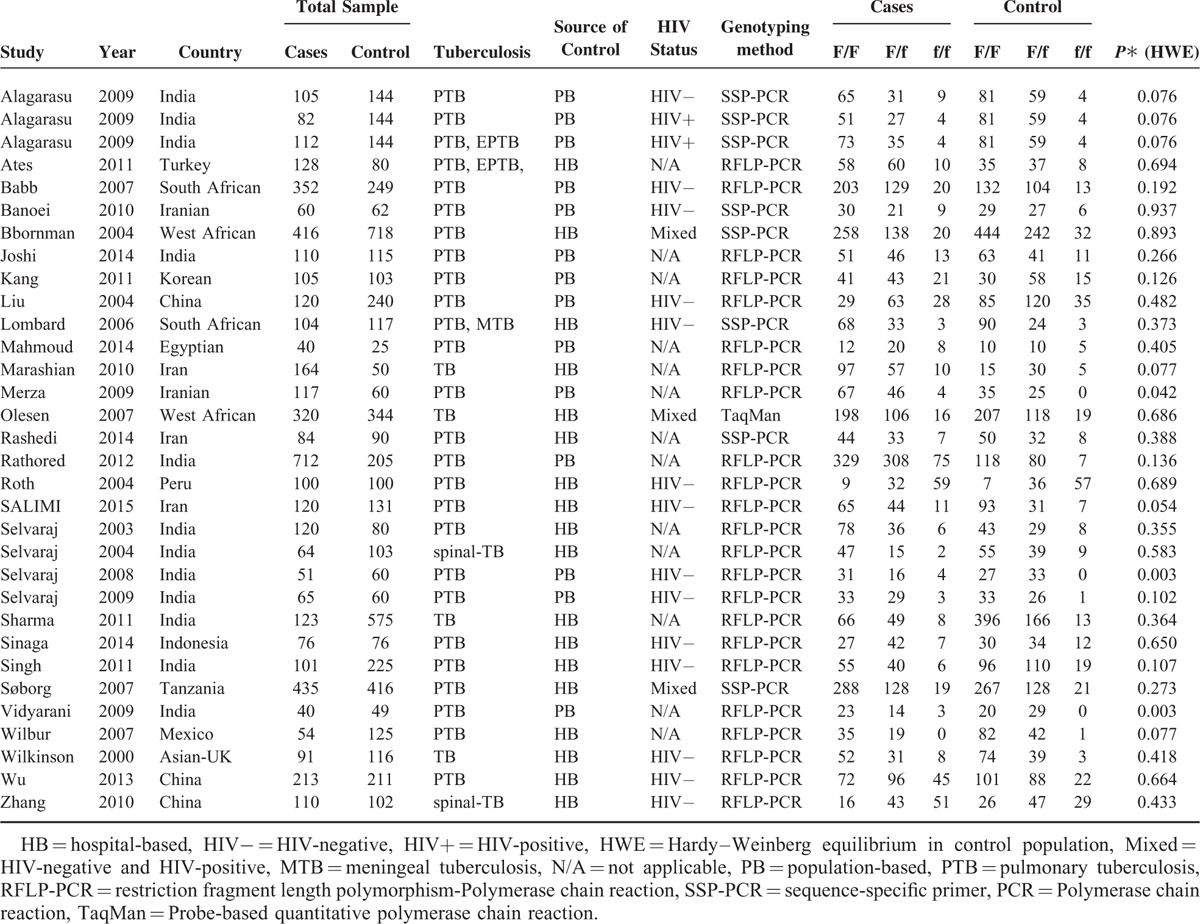
Study Characteristics

### Quantitative Data Synthesis

The evaluations of the association of *Fok*I polymorphisms and TB risk are shown in Table 1. According to the method for dictating the best genetic model,^26^ the estimated OR_1_(ff vs FF), OR_2_(fF vs FF), and OR_3_(ff vs fF) were 1.34 (95% CI = 1.036–1.730), 0.96 (95% CI = 0.827–1.110), and 1.34 (95% CI = 1.122–1.599). These indicated that OR_1_ and OR_3_ were significant (*P* < 0.05) and OR_2_ was not significant (*P* = 0.566); the genetic model was most likely recessive.

**TABLE 1:**
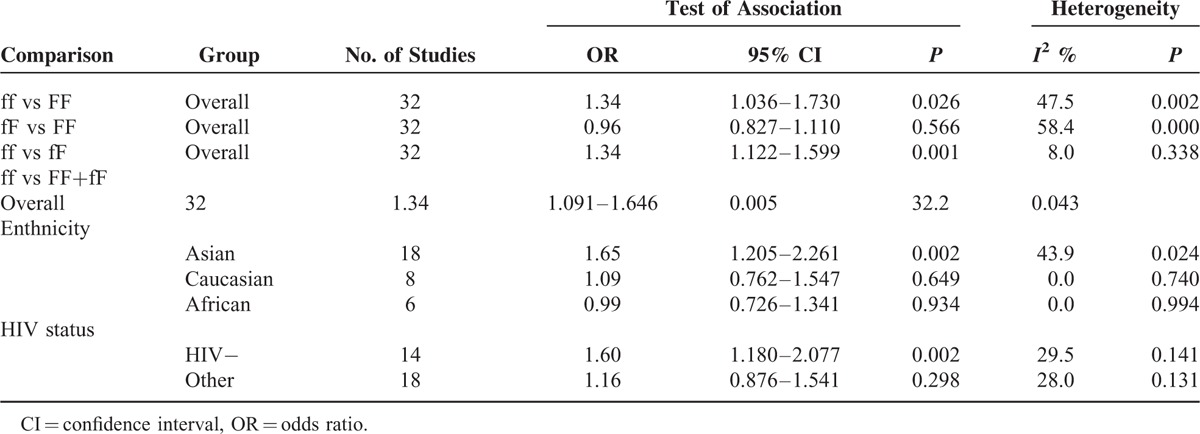
Meta-Analysis of *Fok*I Polymorphism and TB Risk

**FIGURE 2:**
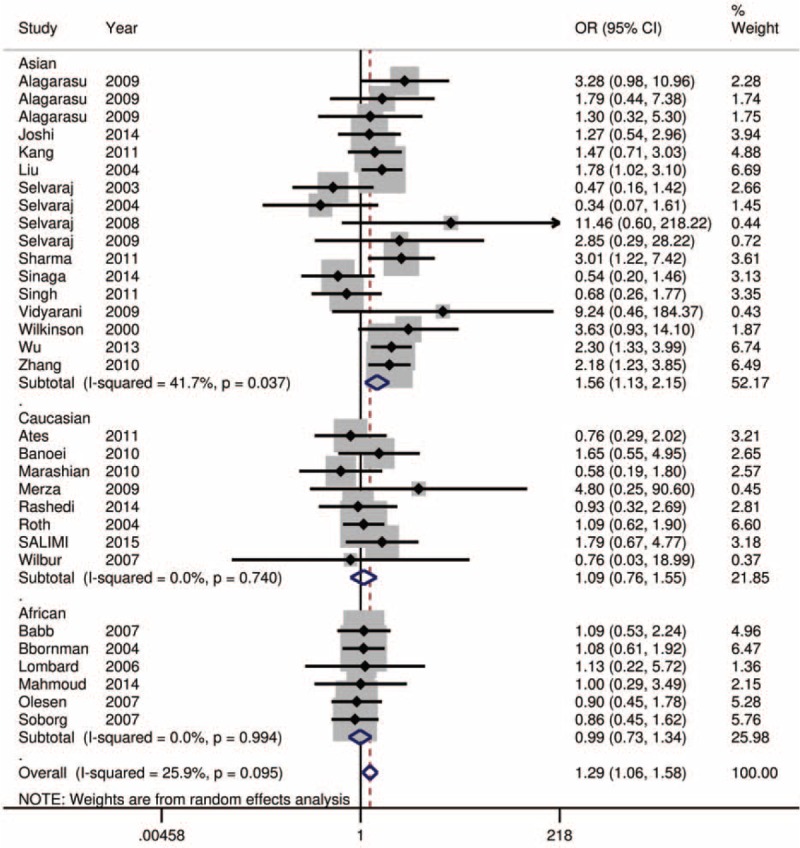
Forest plot for the association between *Fok*I polymorphisms and TB risk stratified by ethnicity in recessive model (ff vs fF+FF).

**FIGURE 3:**
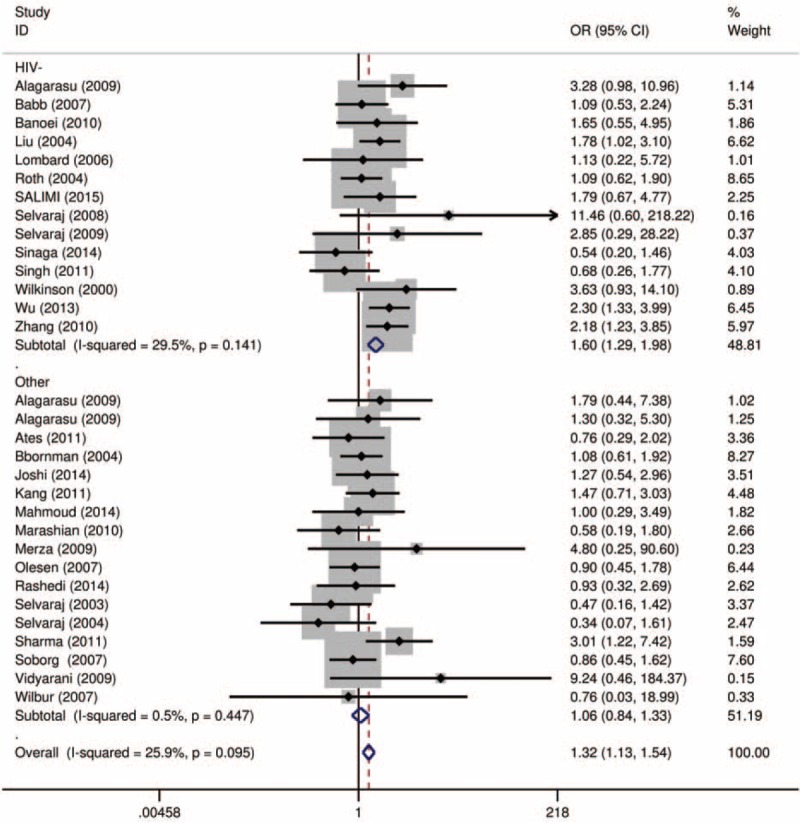
Forest plot for the association between *Fok*I polymorphisms and TB riskstratified by HIV status in recessive model (ff vs fF+FF).

**FIGURE 4:**
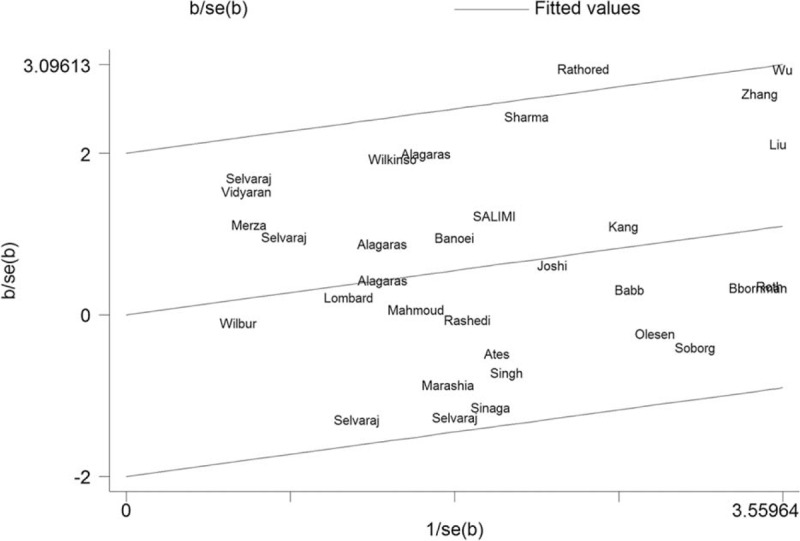
Galbraith plot analysis to evaluate heterogeneity: Rathored et al and Wu et al were the outlier studies in recessive model (ff vs fF+FF).

Using a recessive model, data for the fF and FF group were collapsed and compared to the ff group (ff vs fF+FF). The estimated OR was 1.34 (95% CI = 1.091–1.646, *P* = 0.005). There was moderate heterogeneity in the pooled results (*I*^*2*^ = 32.2%, *P* = 0.043). Therefore, we performed subgroup analysis according to ethnicity and HIV status. In the subgroup analysis by ethnicity (Fig. 2 and Table 1), significant associations were found in the Asian group (OR = 1.65, 95% CI = 1.205–2.261, *P* = 0.002; *I*^*2*^ = 43.9%, and *P* = 0.024 for heterogeneity), but not in the Caucasian group (OR = 1.09, 95% CI = 0.762–1.547, *P* = 0.649; *I*^*2*^ = 0.0%, and *P* = 0.740 for heterogeneity), and the African group (OR = 0.99, 95% CI = 0.726–1.341, *P* = 0.934; *I*^*2*^ = 43.9%, and *P* = 0.024 for heterogeneity). The HIV status was stratified as the HIV-negative TB group and the other group (HIV-positive or no information). As shown in Figure 3 and Table 1, significant associations were found in the HIV-negative TB group (OR = 1.60, 95% CI = 1.180–2.077, *P* = 0.002; *I*^*2*^ = 29.5%, and *P* = 0.141 for heterogeneity). To further explore the sources of heterogeneity, we carried out a Galbraith plot analysis to confirm the outliers that might cause the heterogeneity (Fig. 4). The results showed that Rathored et al^38^ and Wu et al^22^ were the outlier studies. Therefore, we excluded these 2 studies and reran the meta-analysis; the heterogeneity decreased significantly in the recessive model, but the pooled results were not changed significantly (OR = 1.24, 95% CI = 1.016–1.509, *P* = 0.034; *I*^*2*^ = 19.7%, and *P* = 0.170 for heterogeneity).

### Sensitivity Analysis

First, sensitivity analysis was performed by omitting 1 study at a time, and there were no statistically significant changes in all ORs. We then omitted the 3 studies, which were out of HWE, and the statistical significance of the pooled result did not change (OR = 1.31, 95% CI = 1.068–1.604, *P* = 0.010).

### Publication Bias

**FIGURE 5:**
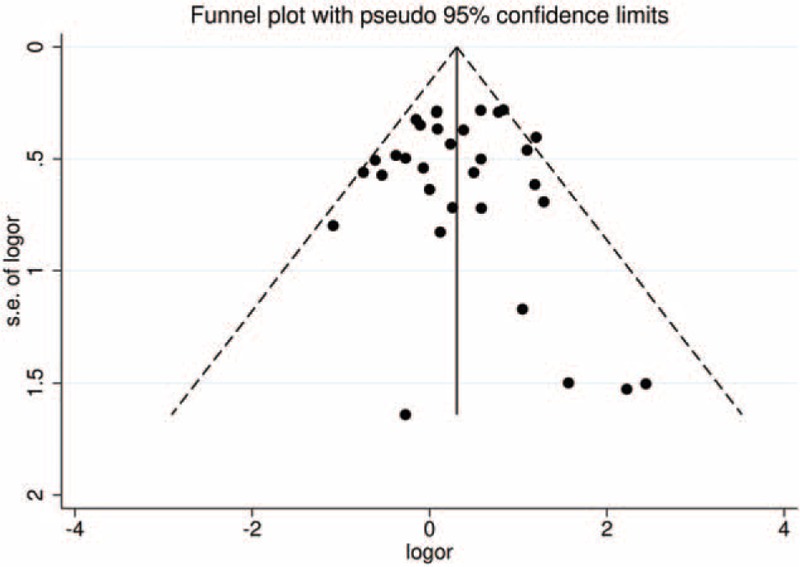
Funnel plot for studies of the association between in recessive model (ff vs fF+FF). The horizontal and vertical axes correspond to the OR and 95% CI. CI = confidence interval; OR = odds ratio.

As shown in Figure 5, the funnel plot was symmetrical. The Begg's funnel plot and the Egger test also confirmed the absence of publication bias among the included studies (*P*_Egger__test_ = 0. 841).

## DISCUSSION

This meta-analysis with 32 case-control studies indicates that VDR *Fok*I polymorphism contributes to the risk of TB. The results suggest that people who had genotype ff had a 34% higher risk of developing TB than people who had genotypes fF/FF, and the risk effect was confirmed in HIV-negative TB patients (OR = 1.60). In addition, results from subgroup analysis stratified by ethnicity indicate that TB risk was increased in Asians with ff genotype (OR = 1.65), but not in Caucasians and Africans.

The results of the present meta-analysis are consistent with a similar meta-analysis performed by Chen et al^16^ in 2013. Compared with the previous study, our meta-analysis included 7 additional studies on the *Fok*I polymorphism.^12,18–23^ Recently, a meta-analysis was performed by Sun et al. However, this meta-analysis missed 11 studies^12,18–20,22,23,39,42–44,47^ according to the specific combinations of search terms and their inclusion and exclusion criteria. In addition, some comparison genetic models in this study were incorrect (eg, the recessive model should be ff vs FF+fF but not ff+fF vs FF). Therefore, this update meta-analysis has more statistical power than the 2 previous studies. Likewise, considering TB is the frequent major opportunistic infection in HIV-infected patients, we carried out a subgroup analysis stratified by HIV status. Interestingly, the risk effect was found only in HIV-negative TB patients. As expected, the heterogeneity decreased significantly, which not only strongly confirms the conclusion that *Fok*I polymorphism contributes to the risk of TB, but also indicates that HIV status was the main source of heterogeneity in the previous meta-analysis. This may be a reason for controversial results from previous studies. Indeed, HIV infection is associated with a greater risk for disease than HIV-negative individuals.^57^ Of note, a study by Xu et al^58^ also focused on this topic; nevertheless, our study is more comprehensive than this study and we found the risk effect only in HIV-negative TB patients but not observed in HIV-positive or not clearly identified group. Therefore, our results suggest it is crucial to avoid selection bias in such genotype association studies.

Our results are also consistent with the functional studies on the VDR genepolymorphisms^59^; the active form of vitamin D (1,25(OH)2D3) is an important immunoregulatory hormone and moves into the nucleus by binding to the VDR complex.^60^ Low vitamin D levels have been found to contribute to the risk of TB infection.^61^ VDR gene polymorphisms are related to vitamin D-related disease,^11^ and significant interaction between vitamin D status and VDR gene polymorphisms has also been observed.^10^ Indeed, VDR polymorphism may influence susceptibility to infectious diseases, such as hepatitis B virus infection^62^ and leprosy.^63^ With respect to *Fok*I polymorphisms, the short 424 amino acid VDR protein variant (corresponding with the C-allele or “big F” allele) has been found to be more active than the long 427 ff variant.^59^ Hence, the f allele of *Fok*I might decrease the activity of the VDR protein, and then block the binding of active vitamin D and VDR. In summary, VDR polymorphism may influence the function of vitamin D and, therefore, contribute to the susceptibility to TB infection.

The present study has some advantages compared with previous studies. First, this update meta-analysis has more statistical power than the 2 previous studies. We also selected the best genetic model to avoid multiple comparisons. Second, we confirmed the conclusion in the HIV-negative TB group, which would further reveal the association between *Fok*I polymorphism and TB. Likewise, our results were relatively reliable for no significant heterogeneity, and some results were given in the sensitivity analysis. However, having some limitations is a required consideration in this study. We should note the potential publication biases when explaining the results, although no significant publication biases were found in this study; positive results mainly come from the Asian region, especially China. In addition, we did not stratify or analyze the other factors, such as sex or clinical and environmental variables, because of a lack of original data from authors. Also, our HIV status-specific analysis included only 2 studies from HIV-positive TB patients, and HIV positive or no information were together as a subgroup in meta-analysis would represent a bias in the analysis and conclusions; additional studies are warranted to explore the relationship between HIV-positive TB and *Fok*I polymorphisms.

## CONCLUSIONS

In conclusion, this meta-analysis confirms that VDR *Fok*I polymorphism contributes to the risk of TB, especially in HIV-negative TB patients and the Asian group. Further studies are required to clarify the role of the *Fok*I polymorphism in HIV-positive TB and in other ethnic groups.
